# *In silico* approach to calculate the transcript capacity

**DOI:** 10.5808/GI.2019.17.3.e31

**Published:** 2019-09-26

**Authors:** Young-Sup Lee, Kyung-Hye Won, Jae-Don Oh, Donghyun Shin

**Affiliations:** Department of Animal Biotechnology, Chonbuk National University, Jeonju 54896, Korea

**Keywords:** fat, genome-wide association study, *in silico* method, transcript capacity, RNA-seq

## Abstract

We sought the novel concept, transcript capacity (TC) and analyzed TC. Our approach to estimate TC was through an *in silico* method. TC refers to the capacity that a transcript exerts in a cell as enzyme or protein function after translation. We used the genome-wide association study (GWAS) beta effect and transcription level in RNA-sequencing to estimate TC. The trait was body fat percent and the transcript reads were obtained from the human protein atlas. The assumption was that the GWAS beta effect is the gene’s effect and TC was related to the corresponding gene effect and transcript reads. Further, we surveyed gene ontology (GO) in the highest TC and the lowest TC genes. The most frequent GOs with the highest TC were neuronal-related and cell projection organization related. The most frequent GOs with the lowest TC were wound-healing related and embryo development related. We expect that our analysis contributes to estimating TC in the diverse species and playing a benevolent role to the new bioinformatic analysis.

## Introduction

There have been various experimental studies regarding enzyme activity [1,2]. Enzyme activity is defined by a measure of the quantity of active enzyme present. Most enzyme activity studies are based on *in vitro* experiments. This approach is a limited method because it does not contain *in vivo* situations. Our approach was to introduce transcript capacity (TC) concept and this concept has both resemblance and difference in comparison to the enzyme activity estimation. It can be considered that TC plays a role like enzyme activity as transcript activity or TC but it is mainly related to the analyzed traits. Thus, TC does not match to the concept of enzyme activity, perfectly. Although enzyme activity is not directly associated with TC, the investigation of TC can be one of the important route to examine enzyme activity because both deal with the capacity of specific cellular units, i.e. enzyme and transcript. We calculated TC using the genome-wide association study (GWAS) beta effect and transcript reads in RNA-sequencing (RNA-seq) data, and our study is based on an *in silico* analysis. Our novel approach was based on that gene effect could be a function of TC and transcript reads. TC refers to the capacity that one unit of transcripts exerts as a cellular function. TC cannot be easily measured in experiments. We calculated TC using bioinformatics studies.

GWAS can be used for finding significant variants and genes associated with given traits. It is a very efficient and powerful method for detecting genes of significance. GWAS has been used to discover disease-associated genes and quantitative trait loci genes [3-5]. Lu et al. [6] tried to discover new loci associated with body fat percent (BF%) and identified cardiometabolic disease risk genomic factors. For TC estimation, we used BF% as the phenotype and performed GWAS. The beta effect in GWAS denotes the coefficient of the regression model and it can be the additive effect of the single nucleotide polymorphism (SNP) in the GWAS [7-9]. The significant markers associated with phenotypes have been used as the significance of the encompassed genes in GWAS. This is plausible because SNPs is linked at those encompassing genes which are usually called “linkage disequilibrium.”

RNA-seq data provides transcripts’ reads [10,11]. Through transcript reads and gene effects, we calculated TC. TC analysis can be important because it can be further analyzed the enzyme activity if the protein expression level instead of transcript reads is given.

## Methods

### Data description

For GWAS, we used Ansan-Anseong cohort data. These were for a study of a chronic diseases within Ansan city and Anseong rural areas in Korea. The dataset comprised men (8,842 people) between 40–69 years of age who had been residents of the region for at least 6 months [12,13]. Our study was from the 3rd Ansan-Anseong cohort dataset version 2.1. The analyzed phenotypes were BF% unit and the covariates were set to be area, age and sex. The SNP dataset was implemented using Affymetrix Genome-wide Human SNP Array 5.0 (Affymetrix, Santa Clara, CA, USA). The mean call rate was 99.01%. The total number of SNPs was 352,228 and after quality control (minor allele frequency < 0.05, Hardy-Weinberg equilibrium p-value < 0.0001 and missing genotype rate > 0.05), 308,003 SNPs were left.

For transcript reads, we used transcript reads per million (TPM) data at the following website (http://www.proteinatlas.org). The Human Protein Atlas was released with protein profile data covering 48 different human tissues and organs, including adipocytes, the kidney and the liver [14,15]. Among these organs, we used adipocyte’s transcript reads data. The TPM data of the Human Protein atlas is based on the reads per gene. Thus the gene length was pre-considered for the accurate reads estimation per gene.

### Genome-wide association study (GWAS)

GWAS was performed using GCTA (a tool for genome-wide complex trait analysis) to estimate the beta effects [9,16]. The following model was:

(1) y=a+bx+g+e      

where *y* is the phenotypic value (BF%), a is the mean term, b is the additive beta effect of the candidate SNP for association, *x* is the SNP genotype indicator variable, *g* is the polygenic effect, and *e* is the residual. The covariates were sex (male and female), area (Ansan and Anseong) and age. The age was factored to be 10-age steps. The *b* was the beta effect of the SNPs.

### TC calculation

TC was calculated using the following relationships:

(2) $$\mathrm{TC}=\frac{\mathrm{effect}\;\mathrm{of}\;\mathrm{gene}}{transcript\;reads}$$

Eq. (2) uses the gene effect and transcript reads (TPM) to determine the TC. We assumed that the effect of the gene was proportional to the transcript reads and TC. The gene effect is proportional to TC as given in Eq. (2). If A and B genes’ effect is 10, 10, respectively and transcript reads are 1,000 and 10,000, then TCs of A and B are 0.01 and 0.001. Thus the capacity of the A transcript is 10-fold stronger than B transcript. One unit of A transcripts influences 10-fold to the traits in comparison to the B transcripts.

### Gene ontology analysis

The gene ontology (GO) analysis was performed using DAVID (Database for Annotation, Visualization and Integrated Discovery) [17]. The gene catalogue was retrieved from Ensembl DB (http://www.ensembl.org). We selected the genes with the highest and lowest TC (top and bottom 5% in TC values) for the GO analysis.

## Results

### Figure description

**Fig. 1.**
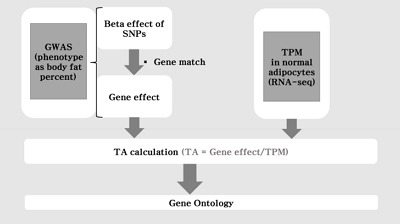
The flow chart of the analysis. The transcript capacity was calculated *in silico* using genome-wide association study (GWAS) and RNA-sequencing (RNA-seq). SNP, single nucleotide polymorphism; TA, transcript activity (synonym: transcript capacity); TPM, transcript per million reads.

**Fig. 2.**
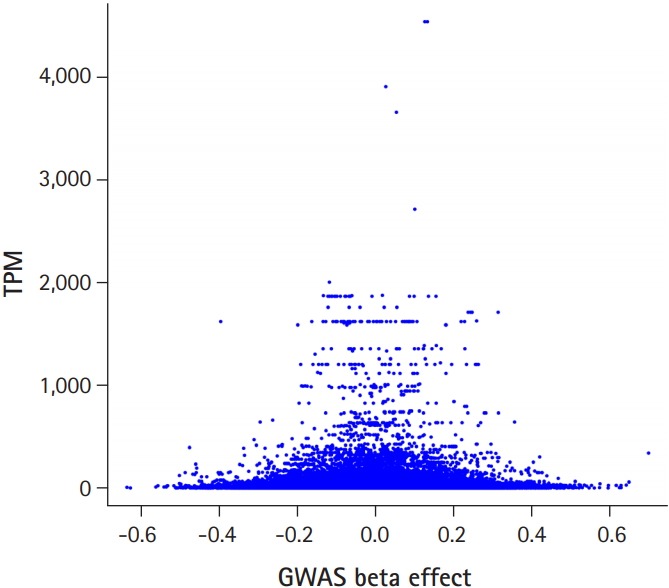
Transcript reads per million (TPM) versus genome-wide association study (GWAS) beta effect. Larger beta effect genes had a smaller TPM across the board.

**Fig. 3.**
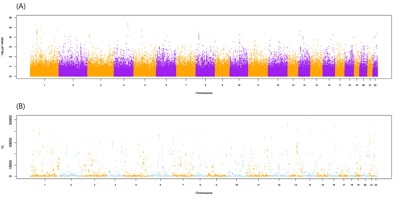
The Manhattan plot of–log_10_(p-value) including total analyzed single nucleotide polymorphisms (SNPs) (A) and transcript capacity (TC) (B) under p-value < 0.05 across chromosomes. The P-value was obtained from the genome-wide association study (GWAS) of the SNPs and those encompassing genes. TC was calculated using the GWAS beta effect and transcript per million reads.

Fig. 1 shows the flow chart of our analysis. It explains the procedure of the TC calculation. The gene effect was calculated using GWAS and TC was calculated using gene effect and TPM. As shown in Eq. (2), the TC unit in our analysis is BF%. Fig. 2 shows the plot of TPM and GWAS beta effect. The genes with a higher TPM had a smaller beta effect across the board. According to Eq. (2), a higher TPM and a smaller beta effect would show a smaller TC. Fig. 3 shows the Manhattan plot of –log_10_(p-value) and the TC across chromosomes. The p-value was from GWAS results and TC was calculated as shown in Fig. 1.

### TC calculation and GO analysis

Table 1 shows the summary statistics (minimum, maximum, average, standard deviation) of GWAS beta effect, TPM and TC. Table 2. shows the gene information with the lowest p-values (<0.0001) and the estimated TC. The TC was simply calculated using Eq. (2). Table 3 illustrates genes’ TC information of the highest p-values (top 5% genes in TC value). The neuronal genes including neuronal-activity regulated genes have important functions in dendrites and synapses and are likely to regulate circuit connectivity directly. Thus for the easy regulation of circuit connectivity, they might have the strategy through possessing low transcript reads and high TC [18]. Brain-derived neurotrophic factor (BDNF; TC, 1.88 BF%) encodes a neurotrophin that is secreted at the synapse. The induction of BDNF promotes both synapse maturation and dendritic growth. BDNF had high TC and its mutation can cause neurological and psychiatric disorders [18]. Table 4 shows the GO with the lowest TC (bottom 5% genes). It shows that the lowest TC’s major GO terms were endoderm formation, wound healing and embryo development. The von Willebrand factor (VWF) is not an enzyme and thus, has no catalytic activity (https://en.wikipedia.org/wiki/Von_Willebrand_factor). In our analysis, the VWF had high transcript reads and low TC (TPM, 155.1; TC, 0.002 BF%).

## Discussion

### GWAS and expression quantitative trait loci

We used the GWAS and RNA-seq data associated with body fat. The expression quantitative trait loci (eQTL) are genomic loci that explain a variation in expression levels of mRNAs (https://en.wikipedia.org/wiki/Expression_quantitative_trait_loci). Parks et al. [19] showed the genetic control of obesity and gut microbiota composition using eQTL analysis. In our analysis, the accurate beta effect estimation accompanied by p-value are crucial and thus eQTL analysis can help better estimating TC because eQTL information contains the mRNA expression level.

### GWAS p-value and accuracy of TC calculation

In GWASs, the significance is guaranteed by the p-value. The p-value is the criteria to dissect the significant variants and those genes from insignificant ones. Although genes’ effects were dissected by the p-value, only the beta effect was used for TC calculation. The gene’s GWAS p-value and beta effect can be varied with respect to analyzed phenotypes. The highly accurate TC calculation should be certified by using diverse phenotypes in GWAS. Additionally, the transcript reads in RNA-seq data can be diverse in various tissues. By using diverse tissues and information from various traits, the accurate TC calculation can be plausible for generally acceptable estimation.

BF% can be used to approximate fat accumulation in adipose tissues. The trait reflects the fat accumulation and the gene’s play in adipose tissue. The GWAS directs the significant variants associated with body fat but the TC quantity only mirrors the transcript activity only related to the analyzed traits as indicated by TC unit (BF%).

Our GWAS calculation for the gene effect was based on the linkage disequilibrium between SNP markers and the gene. Despite the advantages of GWAS using SNP markers, diverse SNP markers per gene can cause a problem. Thus gene-based GWAS can be an another alternative method. And RNA-seq data had better be obtained from the similar sample to the GWAS dataset.

The neuronal and cell projection organization genes were enriched in the GO analysis (Table 2). These genes have low p-values in the GWAS analysis of BF% across the board. Inspecting the relationship between TC and GO terms why the lowest TC values are associated with certain GO terms, is a subject that needs to be addressed. Likewise, the reason why wound healing and embryo development GO terms have low TC values must be elucidated.

### Features of the TC calculation in our study

Unlike previous studies that calculate the protein activity, TC calculation is a high-throughput analysis. Vermeirssen et al.[20] used a quantitative *in silico* analysis to calculate the inhibitory activity of angiotensin I converting enzyme (ACE). They used an *in vivo* analysis to calculate ACE activity, also. Our study to calculate TC is theoretically novel. Additionally, it is feasible not only to calculate TC, but also to calculate other annotated ones including transcription factor whose activity cannot be easily measured experimentally.

## Figures and Tables

**Table 1.** Summary statistics of beta effect, TPM, and TC in our analysis

	Min	Max	Average	SD
Beta	–0.9378	1.326	0.004	0.387
TPM	0.1	1864.5	19.855	70.551
TC	0.000146	7.483	0.371	0.857

Beta, GWAS beta effect; TPM, transcript per million reads; TC, transcript capacity.

**Table 2.** Genes with the lowest p-values (cutoff 0.0001) and their TC estimation

CHROM	SNP	Position	Beta	p-value	Gene	TPM	TC
1	SNP_A-2261105	56496439	–0.49	0.00000569	*PLPP3*	147.9	0.003
1	SNP_A-2137301	77704683	0.39	0.00000908	*USP33*	45	0.009
4	SNP_A-4203089	134200024	0.4	0.0000107	*PABPC4L*	1.6	0.25
5	SNP_A-1821378	75406064	0.43	0.0000138	*COL4A3BP*	37.7	0.01
1	SNP_A-1848974	56496993	–0.46	0.0000169	*PLPP3*	147.9	0.003
1	SNP_A-2302611	56520770	–0.38	0.0000176	*PLPP3*	147.9	0.003
1	SNP_A-2276931	235264645	0.38	0.0000191	*ARID4B*	12.7	0.03
5	SNP_A-4258112	75370070	0.43	0.0000201	*COL4A3BP*	37.7	0.01
5	SNP_A-4193701	75413307	0.42	0.0000233	*COL4A3BP*	37.7	0.01
14	SNP_A-2203004	32144721	0.54	0.0000247	*ARHGAP5*	26.9	0.02
5	SNP_A-2111116	171218237	–0.42	0.0000335	*RANBP17*	0.2	2.12
22	SNP_A-2212209	42624987	0.7	0.0000391	*CYB5R3*	337.7	0.002
11	SNP_A-1946877	114585252	0.36	0.000041	*NXPE4*	0.1	3.6
14	SNP_A-2203008	32145585	0.41	0.0000533	*ARHGAP5*	26.9	0.015
10	SNP_A-4304897	77131018	–0.46	0.0000558	*KCNMA1*	11.1	0.04
16	SNP_A-2161617	78907824	–0.45	0.0000603	*WWOX*	10.5	0.04
6	SNP_A-4211857	96568889	0.58	0.0000811	*FHL5*	41.6	0.014
2	SNP_A-2266417	75681594	–0.35	0.0000813	*MRPL19*	35	0.01
2	SNP_A-2266417	75681594	–0.35	0.0000813	*GCFC2*	10.1	0.03
2	SNP_A-4257172	46029907	0.38	0.000082	*PRKCE*	5	0.08
5	SNP_A-1943727	75381858	0.4	0.0000879	*COL4A3BP*	37.7	0.01
5	SNP_A-2283204	75433640	0.39	0.0000927	*COL4A3BP*	37.7	0.01
1	SNP_A-1827525	216928377	–0.34	0.0000937	*ESRRG*	0.4	0.85

The estimation of TC and TPM belongs to the corresponding gene.

TC, transcript capacity; CHROM, chromosome name; SNP, single nucleotide polymorphism; TPM, transcript reads per million.

**Table 3.** GO for the highest (as TC values) top 1% of the genes

Term	Count	p-value	Genes	Fold enrichment
GO:0007399~nervous system development	29	0.000000124	*OPRM1, RP1, DPF3, GRIK1, GRIP1, SYT2, CTNND2, RP1L1, RIMS2, BDNF, BARX2, KIRREL3, RFX4, MDGA2, GRIN2A, ARMC4, RPGRIP1, ELAVL4, ALK, GRHL2, PPP1R17, CTNNA2, EPHA7, HYDIN, CHRM3, RASGRF1, CHRM2, CHST8, ATP8A2*	2.89
GO:0048858~cell projection morphogenesis	16	0.00000257	*RP1, RFX4, SYT2, RP1L1, CTNND2, ARMC4, ELAVL4, RIMS2, DNAH7, NME8, CTNNA2, EPHA7, BDNF, HYDIN, ATP8A2, KIRREL3*	4.34
GO:0032990~cell part morphogenesis	16	0.00000349	*RP1, RFX4, SYT2, RP1L1, CTNND2, ARMC4, ELAVL4, RIMS2, DNAH7, NME8, CTNNA2, EPHA7, BDNF, HYDIN, ATP8A2, KIRREL3*	4.23
GO:0030030~cell projection organization	20	0.00000418	*RP1, MYO1A, RFX4, GRIP1, SYT2, RP1L1, CTNND2, ARMC4, ACTN2, ELAVL4, RIMS2, DNAH7, NME8, CTNNA2, BDNF, EPHA7, HYDIN, RASGRF1, ATP8A2, KIRREL3*	3.32
GO:0032989~cellular component morphogenesis	18	0.000075	*RP1, RFX4, SYT2, RP1L1, CTNND2, ARMC4, ACTN2, ELAVL4, RIMS2, DNAH7, NME8, GRHL2, CTNNA2, BDNF, EPHA7, HYDIN, ATP8A2, KIRREL3*	2.95
GO:0035082~axoneme assembly	5	0.0000991	*RP1, HYDIN, RP1L1, ARMC4, DNAH7*	20.4
GO:0048666~neuron development	15	0.000104	*RP1, GRIP1, SYT2, RP1L1, CTNND2, RPGRIP1, ELAVL4, RIMS2, ALK, CTNNA2, EPHA7, BDNF, RASGRF1, ATP8A2, KIRREL3*	3.36
GO:0000902~cell morphogenesis	17	0.000123	*RP1, RFX4, SYT2, RP1L1, CTNND2, ARMC4, ELAVL4, RIMS2, DNAH7, NME8, GRHL2, CTNNA2, BDNF, EPHA7, HYDIN, ATP8A2, KIRREL3*	2.96
GO:0022008~neurogenesis	18	0.000227	*OPRM1, RP1, GRIP1, SYT2, MDGA2, GRIN2A, RP1L1, CTNND2, RPGRIP1, ELAVL4, RIMS2, ALK, CTNNA2, BDNF, EPHA7, RASGRF1, ATP8A2, KIRREL3*	2.69
GO:0048699~generation of neurons	17	0.000346	*OPRM1, RP1, GRIP1, SYT2, MDGA2, RP1L1, CTNND2, RPGRIP1, ELAVL4, RIMS2, ALK, CTNNA2, BDNF, EPHA7, RASGRF1, ATP8A2, KIRREL3*	2.71
GO:0042384~cilium assembly	7	0.000355	*RP1, HYDIN, RFX4, RP1L1, ARMC4, DNAH7, NME8*	7.35
GO:0030182~neuron differentiation	16	0.00037	*RP1, GRIP1, SYT2, MDGA2, RP1L1, CTNND2, RPGRIP1, ELAVL4, RIMS2, ALK, CTNNA2, EPHA7, BDNF, RASGRF1, ATP8A2, KIRREL3*	2.82
GO:0010927~cellular component assembly involved in morphogenesis	8	0.000385	*RP1, HYDIN, RFX4, RP1L1, ARMC4, ACTN2, DNAH7, NME8*	5.88
GO:0048468~cell development	21	0.000399	*OPRM1, RP1, IL5, GRIP1, SYT2, RP1L1, CTNND2, ACTN2, RPGRIP1, ELAVL4, RIMS2, ALK, GRHL2, CTNNA2, BDNF, EPHA7, HYDIN, RASGRF1, ATP8A2, NUP210L, KIRREL3*	2.31
GO:0007268~chemical synaptic transmission	11	0.000405	*OPRM1, CDH8, KCNQ5, CHRM3, GRIK1, RASGRF1, CHRM2, SYT2, GRIN2A, RIMS2, OPRD1*	3.94
GO:0098916~anterograde trans-synaptic signaling	11	0.000405	*OPRM1, CDH8, KCNQ5, CHRM3, GRIK1, RASGRF1, CHRM2, SYT2, GRIN2A, RIMS2, OPRD1*	3.94
GO:0099536~synaptic signaling	11	0.000405	*OPRM1, CDH8, KCNQ5, CHRM3, GRIK1, RASGRF1, CHRM2, SYT2, GRIN2A, RIMS2, OPRD1*	3.94
GO:0099537~trans-synaptic signaling	11	0.000405	*OPRM1, CDH8, KCNQ5, CHRM3, GRIK1, RASGRF1, CHRM2, SYT2, GRIN2A, RIMS2, OPRD1*	3.94
GO:0001578~microtubule bundle formation	5	0.000443	*RP1, HYDIN, RP1L1, ARMC4, DNAH7*	13.86
GO:0044782~cilium organization	7	0.000593	*RP1, HYDIN, RFX4, RP1L1, ARMC4, DNAH7, NME8*	6.67
GO:0030031~cell projection assembly	9	0.000636	*RP1, MYO1A, HYDIN, RFX4, RP1L1, ARMC4, ACTN2, DNAH7, NME8*	4.64
GO:0060271~cilium morphogenesis	7	0.000922	*RP1, HYDIN, RFX4, RP1L1, ARMC4, DNAH7, NME8*	6.13

The major gene ontology (GO) terms were neuronal and cell projection organization-related.

TC, transcript capacity.

**Table 4.** GO for the lowest (as TC values) bottom 1% of the genes

Term	Count	p-value	Gene	Fold enrichment
GO:0007369~gastrulation	6	0.0000374	*COL4A2, DLD, LAMB1, MMP2, PLPP3, FN1*	15.22
GO:0042060~wound healing	7	0.0000543	*VWF, CD34, HMOX1, IGF1, MYH9, PLPP3, FN1*	9.98
GO:0009611~response to wounding	7	0.000136	*VWF, CD34, HMOX1, IGF1, MYH9, PLPP3, FN1*	8.45
GO:0009790~embryo development	10	0.000165	*FKBP8, COL4A2, DLD, IGF1, LAMB1, MYH9, MMP2, PLPP3, RCN1, FN1*	4.69
GO:0035987~endodermal cell differentiation	4	0.000206	*COL4A2, LAMB1, MMP2, FN1*	33.52
GO:0001944~vasculature development	8	0.000217	*COL4A2, ANP32B, CD34, QKI, MYH9, MMP2, PLPP3, THY1*	6.2
GO:0031589~cell-substrate adhesion	6	0.000273	*VWF, CD34, FERMT2, LAMB1, THY1, FN1*	9.99
GO:0001706~endoderm formation	4	0.000366	*COL4A2, LAMB1, MMP2, FN1*	27.65
GO:0007492~endoderm development	4	0.000751	*COL4A2, LAMB1, MMP2, FN1*	21.69

The major gene ontology (GO) terms were wound-healing and embryo development.

TC, transcript capacity.
